# A systematic review and meta‐analysis of the prevalence of human cytomegalovirus shedding in seropositive pregnant women

**DOI:** 10.1002/rmv.2399

**Published:** 2022-10-05

**Authors:** Shari Sapuan, Anastasia A. Theodosiou, Blair L. Strang, Paul T. Heath, Christine E. Jones

**Affiliations:** ^1^ St George's, University of London Centre for Neonatal and Paediatric Infection London UK; ^2^ University of Southampton Clinical and Experimental Sciences Southampton UK; ^3^ St George's, University of London Institute for Infection and Immunity London UK; ^4^ Faculty of Medicine and Institute for Life Sciences University of Southampton and NIHR Southampton Clinical Research Facility and NIHR Southampton Biomedical Research Centre University Hospital Southampton NHS Foundation Trust Southampton UK

**Keywords:** cytomegalovirus, pregnant, prevalence, seropositive, shedding

## Abstract

The detection of human cytomegalovirus (HCMV) in an individual's bodily fluid by culture techniques or through HCMV DNA detection by polymerase chain reaction, is known as HCMV shedding. Human cytomegalovirus shedding has the potential to transmit HCMV infection, where an individual can become infected with HCMV through contact with the bodily fluid of another individual containing HCMV. Human cytomegalovirus shedding can occur in primary infection and in non‐primary infection for individuals with prior infection (HCMV seropositive). Human cytomegalovirus infection causes few or no symptoms in a pregnant woman, but can cause significant harm to her foetus if congenital CMV (cCMV) infection occurs. The association between HCMV shedding in HCMV seropositive pregnant women and the vertical transmission of HCMV to result in cCMV infection is poorly investigated, challenged by a limited understanding of the distribution of HCMV shedding in HCMV seropositive pregnant women. We systematically reviewed the published literature to describe the prevalence of HCMV shedding in HCMV seropositive women during pregnancy up to delivery. This analysis identified nine studies that met our eligibility criteria. In these studies, the prevalence of HCMV shedding in any bodily fluid of HCMV seropositive women during pregnancy and at delivery ranged from 0% to 42.5%. A meta‐analysis, performed on six of the nine studies with suitable sample sizes, estimated a pooled prevalence of 21.5% [95% CI 12.7%,30.3%]. To our knowledge, this is the first review to systematically search the literature to summarise the prevalence of HCMV shedding in HCMV seropositive pregnant women. These estimates can help in the development of disease burden models and therapeutic or preventative strategies against cCMV infection in the context of non‐primary maternal HCMV infection.

**Abbreviations**
B: blood
C: cervical secretions
cCMV: congenital cytomegalovirus
CI: confidence interval
CMV: cytomegalovirus
DNA: deoxyribonucleic acid
GW: gestation in weeks
HCMV: human cytomegalovirus
IgG: immunoglobulin G
IgM: immunoglobulin M
MS: multiple sites
MT: multiple time‐points
PCR: polymerase chain reaction
RR: risk ration
S: saliva
SS: single site
ST: single time‐point
T: trimester
U: urine
USA: United States of America
V: vaginal secretions

## 1 INTRODUCTION

Following primary infection, human cytomegalovirus (HCMV) can establish life‐long latency in an infected individual and may reactivate, known as non‐primary HCMV infection.^1^ Non‐primary HCMV infection can also occur from reinfection with a different HCMV strain.^1^ The current standard for diagnosing primary HCMV infection is the detection of HCMV immunoglobulin G (IgG) with or without HCMV immunoglobulin M (IgM) in an individual with previously undetectable HCMV IgG and IgM, known as seroconversion.^2^ The diagnosis of non‐primary HCMV infection in an individual with prior infection, known as being HCMV seropositive, cannot therefore rely on conventional serological findings alone.^2^

The detection of HCMV in an individual's bodily fluid by culture techniques or through HCMV DNA detection by polymerase chain reaction (PCR), is known as HCMV shedding.^3^ HCMV shedding may suggest that an individual is acutely infected with HCMV, where both primary and non‐primary HCMV infection can result in excretion of viral particles into the individual's bodily fluid.^1^, ^2^ Furthermore, HCMV shedding has the potential to transmit HCMV infection, where an individual can become infected with HCMV through contact with bodily fluid of another individual containing HCMV.^1^, ^2^ Young children, who are known to shed HCMV in their bodily fluids for prolonged periods of time, are a common source of HCMV transmission, including to pregnant women.^1^, ^3^

The extent of HCMV disease largely depends on the individual's immune response. Most healthy individuals, including pregnant women, will have mild or no symptoms.^1^, ^2^, ^3^ However, HCMV infection can cause severe symptoms in an immunocompromised individual (such as in a HIV‐positive individual or an organ or haematopoietic stem cell transplant recipient), as well as in an infant when congenital CMV (cCMV) infection is acquired before birth via vertical transmission from a pregnant woman infected with HCMV.^1^, ^2^

Congenital CMV infection is the most common congenital infection in the UK and worldwide.^4^, ^5^ Up to 25% of children with cCMV infection will have long‐term neurodevelopmental impairments, the most common being sensorineural hearing loss.^6^, ^7^ Learning disability, behavioural problems, visual impairment, cerebral palsy, epilepsy and autism spectrum disorder have also been associated with cCMV infection.^6^, ^7^, ^8^ Children with cCMV infection may require significant input from health and social care services, which can have a large impact on families and is associated with a significant cost to society.^8^, ^9^

Due to the challenges of diagnosing non‐primary HCMV infection serologically, the detection of HCMV shedding by culture or PCR in HCMV seropositive pregnant women may offer a suitable diagnostic approach. This may be a valuable tool for the evaluation of preventative and therapeutic strategies, such as vaccine development and risk‐reduction measures against cCMV infection in the context of non‐primary maternal HCMV infection.

The association between HCMV shedding in HCMV seropositive pregnant women and the vertical transmission of HCMV to result in cCMV infection is poorly investigated, challenged by a limited understanding of the distribution of HCMV shedding in HCMV seropositive pregnant women. To facilitate the estimation of disease burden which can contribute towards the development of preventative and therapeutic strategies for cCMV infection in the context of non‐primary maternal infection, we analysed the published literature to understand the prevalence of HCMV shedding in HCMV seropositive immune‐competent women during pregnancy up to delivery.

## 2 MATERIALS AND METHODS

### 2.1 Search strategy

The systematic review protocol, conduct and report, were executed in accordance with the criteria set out by the Preferred Reporting Items for Systematic Reviews and Meta‐Analyses guidelines.^10^, ^11^ International prospective register of systematic reviews (PROSPERO)^12^ registration number: CRD42021275205.

We developed a search strategy with guidance from an academic liaison librarian and a Cochrane information specialist at St George's, University of London. The literature search was conducted on 7 December 2021 on three electronic bibliographic databases; MEDLINE, Embase, and Web of Science Core Collection. No time limit to the publication was set. The following search terms were used in different combinations: cytomegalovirus, CMV, shedding, excretion, replication, DNA, PCR, culture, seropositive, IgG, pregnant, and maternal. The search terms were adapted for use with the bibliographic database (full search strategy available under Supplementary Material).

The *CoCoPop* mnemonic (condition, context, and population) was used to determine the inclusion criteria^13^ for the studies:

*Condition*: HCMV shedding, defined as the detection of HCMV by culture techniques or the detection of HCMV DNA by PCR, in any maternal bodily fluid.
*Context*: HCMV seropositivity (HCMV IgG detected, IgM not detected), prior to or at the start of HCMV shedding assessment, with no suspicion of or confirmed primary HCMV infection.
*Population*: Pregnant women of any age and ethnicity, at any trimester including at delivery, and in any country. Studies where only pregnant women with confirmed or suspected immunosuppressive, immunocompromised or immunodeficient condition or status were excluded.

Observational studies including cohort, cross‐sectional and case‐control studies were included. Experimental studies, case reports, case series, review articles, full articles not in English, conference abstract and unpublished studies were excluded.

Identified studies were screened, deduplicated and catalogued on Mendeley Reference Manager. Title and abstract screening using the search strategy outlined above was performed independently by two‐blinded reviewers (S.S., A.T.) using Rayyan Qatar Computing Research Institute. The full‐text articles were also reviewed in a blinded manner by the same authors. Any disagreements were resolved through arbitration from the third reviewer (C.J.).

### 2.2 Data extraction

Data were extracted independently by the first author (S.S.) and reviewed by the second author (A.T.) using a standardised data extraction template. Any disagreements were resolved through arbitration from a third author (C.J.). Data extracted on demographics included: country, study type, setting, age, ethnicity, information on children, and sample size. Data extracted on HCMV shedding included: bodily fluid site(s), time‐point(s), detection method(s), prevalence in any bodily fluid, and prevalence by specific bodily fluid site(s).

Two authors (S.S. and A.T.) independently reviewed the studies that were included in this review and performed independent critical appraisal of the included studies using the *Joanna Briggs Institute Critical Appraisal Checklist for Prevalence Studies 2013* ^13^ to assess the methodological quality and to determine the extent to which the studies have addressed the possibility of bias in its design, conduct and analysis. The results of this appraisal did not preclude the studies from being included in the review but were used to inform synthesis and interpretation of the study results.

### 2.3 Statistical analysis

A narrative synthesis of the included studies, structured around the condition, context and population specified and the quality assessment, was produced. Individual study characteristics structured around the data extraction categories were summarised in a descriptive table. Meta‐analysis of the prevalence of HCMV shedding in HCMV seropositive pregnant women, overall, and separately by bodily fluid site where at least two studies reported prevalence data by site that could be extracted, was performed to provide a summary estimate on the global burden of HCMV shedding prevalence in HCMV seropositive pregnant women. The meta‐analysis was performed using Microsoft Excel,^14^ grouped by random‐effects model with 95% CI to allow for between‐study variation, and to adjust the weight of each study based on the assumption that observed variability is due to both sampling error and true variability in the population.^13^, ^14^ Analysis of heterogeneity across the studies was performed through the calculation of Higgins I^2^ (I^2^) to assess the extent of inconsistency of findings across the studies included in the meta‐analysis.^13^, ^14^ The minimum sample size for studies to include in the meta‐analysis was 94, calculated based on the adequate sample size in prevalence study formula^15^; *n* = *[Z* ^*2*^ *P(1−P)]/d* ^*2*^ (*Z* = 1.96, *p* = 0.425, *d* = 0.10). The prevalence estimated from the study with the highest prevalence was used in the prevalence study formula as the expected prevalence was not known. A forest plot with a pooled estimate of the effect sizes^14^ (prevalence of HCMV shedding in HCMV seropositive pregnant women overall) was created.

## 3 RESULTS

### 3.1 Systematic review study selection

**FIGURE 1:**
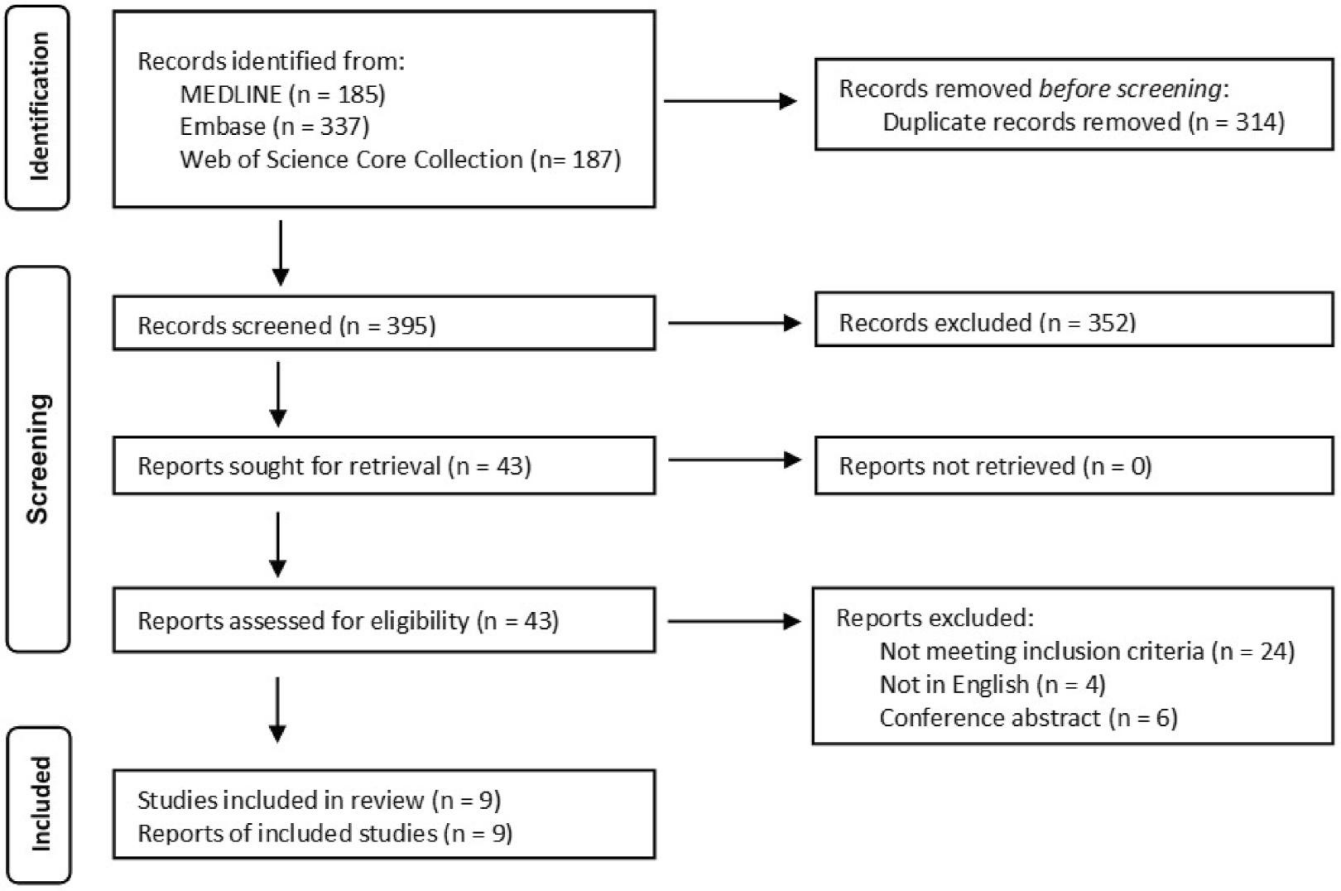
Flow diagram of the identification of studies for the systematic review. Flow diagram of the identification of studies of the systematic review, adapted from PRISMA 2020 flow diagram^11^ for systematic review. Studies were identified according to the search strategy and inclusion criteria developed for the review from three databases: MEDLINE, Embase, and Web of Science Core Collection.

We conducted a systematic literature search to identify studies detailing the prevalence of HCMV shedding in HCMV seropositive pregnant women. Out of 709 articles generated from our literature search, 395 articles were selected for abstract evaluation following deduplication (Figure 1). Of the 395 articles, 43 studies were screened for full‐text evaluation, and nine studies met the inclusion criteria (Figure 1). Three studies appeared to have met the inclusion criteria but were excluded: the seropositive status of the pregnant women with HCMV shedding was unclear in two studies,^16^, ^17^ and the source of shedding was of a foetal bodily fluid for one study.^18^

A description of the nine studies included in the systematic review derived from the data extraction is shown in Table 1. Only data that met the inclusion criteria from included studies are stated, data not meeting the inclusion criteria from these same studies are not stated.

**TABLE 1:** Characteristics of included studies derived from the data extraction

No	Study	Study design	Country	Setting	Demographic a. Age (years) b. Ethnicity c. Data on children reported	Size	HCMV shedding site(s)	HCMV shedding time‐point(s)	HCMV shedding design	HCMV shedding method	HCMV shedding prevalence overall (% [95% CI] (x/n))	HCMV shedding prevalence by site (Site: % [95% CI] (y/n))	Quality assessment score*
1	Gehrz 1981^19^	Cohort study	USA	Unknown	Unknown Unknown No	8	U, C	1^st^ T, 2^nd^ T, 3^rd^ T, at delivery	MS, MT	Culture	0% [0%,36.9%] (0/8)	U: 0% [0%,36.9%] (0/8) C: 0% [0%,36.9%] (0/8)	22% (low)
2	Faix 1983^20^	Single‐centre cohort study	USA	Pregnant women attending one obstetric clinic for adolescents	Range 13–19 White, black No	45	U, C, S, B	1^st^ prenatal visit, <20 GW, 20–30 GW, at delivery	MS, MT	Culture	35.6% [21.9%,51.2%] (16/45)	U: 13.3% [5.1%,26.8%] (6/45) C: 22.2% [11.2%,37.1%] (10/45) S: 8.9% [2.5%,21.2%] (4/45) B: 2.2% [0.1%,11.8%] (1/45)	44% (moderate)
3	Chandler 1985^21^	Cohort study	USA	Pregnant women attending their first prenatal clinic	Mean 24 White, black, Asian, Mexican American, native American, mixed No	564	C	1^st^ prenatal clinic visit (mean 14.3 GW)	SS, ST	Culture	14.0% [11.3%,17.2%] (79/564)	C: 14.0% [11.3%,17.2%] (79/564)	89% (high)
4	Shen 1993^22^	Multi‐centre cohort study	Taiwan	Pregnant women attending two obstetric clinics	<26, 26–35, >36 (no range) Unknown Yes	350	U, C	14–16 GW, 24–26 GW	MS, MT	CMV DNA PCR	27.1% [22.6%,32.1%] (95/350)	U: 11.1% [8.0%,14.9%] (39/350) C: 20.6% [24.0%,36.5%] (66/220)	72% (high)
5	Kyriazopoulou 1996^23^	Cohort study	Greece	Unknown	Unknown Unknown No	32	B	17–20 GW	SS, ST	CMV DNA PCR	0% [0%,10.9%] (0/32)	B: 0% [0%,10.9%]϶(0/32)	17% (low)
6	Sahiner 2015^24^	Cohort study	Turkey	Pregnant women planned for elective C‐section	Mean 30 Unknown No	101	U, B	Just before delivery	MS, ST	CMV DNA PCR	5.5% [2.2%,12.5%] (6/101)	U: 5.0% [1.6%.11.2%] (5/101) B: 2.0% [0.2%,7.0%] (2/101)	39% (low)
7	Mujtaba 2016^25^	Multi‐centre cohort study	Pakistan	Pregnant women seeking antenatal care at two hospitals	Mean 27 Unknown No	347	B	1^st^ ‐ 3^rd^ T	SS, ST	CMV DNA PCR	9.2% [6.4%,12.8%] (32/347)	B: 9.2% [6.4%,12.8%] (32/347)	67% (moderate)
8	Barbosa 2018^26^	Multi‐centre cohort study	Brazil	Pregnant women attending first antenatal visit at six centres	Range 14–41 (mean 24) White, black, mixed black Yes	120	U, S, V, B	1^st^ T, 2^nd^ T, 3^rd^ T	MS, MT	CMV DNA PCR	30.8% [23.3%,39.6%] (37/120)	S: 18.3% [11.9%,26.4%] (22/120) U: 10.8% [5.9%,17.8%] (13/120) V: 5.8% [2.4%,11.7%] (7/120) B: 0.8% [0%,4.6%] (1/120)	61% (moderate)
9	Zelini 2021^27^	Single‐centre cohort study	Italy	Pregnant women attending first antenatal visit at one centre	Range 19–49 Unknown Yes	228	U, S, V, B	9–16 GW, 17–25 GW, 26–37 GW, at delivery	MS, MT	CMV DNA PCR	42.5% [36.0%,49.2%] (97/228)	Unknown	78% (high)

*Note*: Table detailing the characteristic of included studies ordered by year of publication in ascending order. Only data that met the inclusion criteria from included studies is stated, data not meeting the inclusion criteria from these same studies are not stated.

Abbreviations: B, blood; C, cervical secretions; CI, confidence interval; DNA, deoxyribonucleic acid; GW, gestation in weeks; MS, multiple sites; MT, multiple time‐points; n, included population; PCR, polymerase chain reaction; S, saliva; SS, single site; ST, single time‐point; T, trimester; U, urine; V, vaginal secretions; x, no. of women with HCMV shedding in at least one site during at least one time‐point; y, no. of women with HCMV shedding in the specific site during at least one time‐point.

*Average score of the critical appraisal performed by two authors using the Joanna Briggs Institute Critical Appraisal Checklist for Prevalence Studies 2013.^13^

### 3.2 Design and methods of selected studies for analysis

All studies included in the systematic review employed a cohort study design, with a sample size ranging between 8 and 564 women (Table 1). Across the studies, the bodily fluid sites for HCMV shedding detection were saliva, urine, vaginal secretions, cervical secretions, and blood. Six studies (Table 1; No. 1, 2, 4, 6, 8, 9) adopted sample collection from at least two (range 2–4) sites, and three studies (Table 1; No. 3, 5, 7) from a single site. Five studies (Table 1; No. 1, 2, 4, 8, 9) involved sample collection at multiple (range 2–4) time‐points, and four studies (Table 1; No 3, 5, 6, 7) at a single time‐point. Six studies (Table 1; No. 3–8) included sample collection from women only during pregnancy, and three (Table 1; No. 1, 2, 9) also included collection at delivery. Timing of sampling varied between the studies, with some reporting sampling time‐points according to trimester of pregnancy, range of gestational weeks, or proximity to (just before or just after) delivery. Five studies (Table 1; No. 1, 2, 4, 8, 9) reported at least two (range 2–4) sampling time‐points, and four (Table 1; No. 3, 5–7) reported a single time‐point. Six studies (Table 1; No. 4–9) used PCR to detect HCMV DNA as the method of HCMV shedding assessment, and three (Table 1; No. 1–3) used viral culture techniques.

### 3.3 Demographics of individuals in selected studies for analysis

The nine studies in the systematic review represented four continents: three from North America, three from Asia, two from Europe and one from South America. Reporting of population age varied across studies, with mean age reported in four studies (Table 1; No. 3, 6–8), age range in three studies (Table 1; No. 2, 8, 9), and age category in one study (Table 1; No. 4); in two studies (Table 1; No. 1, 5), age was either not reported or it was not possible to extract the relevant data. Data on the HCMV seropositive pregnant women's living children in relation to the HCMV shedding assessment was only reported in three studies (Table 1; No. 4, 8, 9) which were also varied. *Shen et al.* (Table 1; No. 4) found no difference between groups of women who had children attending nursery or kindergarten and those who did not in the outcome of HCMV shedding in urine (11.8% vs. 10.8%, *p*‐value 0.83) and in cervical secretions (28.0% vs. 30.6%, *p*‐value 0.73). *Barbosa et al.* (Table 1; No. 8) reported that pregnant women living with or providing daily care to children aged 3–6 years old were twice as likely to shed HCMV in any site as those not living with or providing daily care to children of this age (58% vs. 26%: adjusted RR 2.21 [95% CI 1.37,3.56]). *Zelini et al.* (Table 1; No. 9) reported on whether participants had children aged less than 36 months or not and found no difference in HCMV shedding between these groups (28.6% vs. 20.7%, *p*‐value 0.18).

### 3.4 Prevalence of human cytomegalovirus shedding in selected studies for analysis

The prevalence of HCMV shedding in pregnant women, at least once in any bodily fluids and at any point during pregnancy up to delivery, ranged from 0% to 42.5%. The highest prevalence was reported by *Zelini et al.* (Table 1; No. 9), and the lowest by *Gehrz et al.* (Table 1; No 1) and *Kyriazopoulou et al.* (Table 1; No.5), where both were of a sample size below the minimum sample size deemed suitable for a meta‐analysis for a pooled prevalence estimate. We were able to extract data on prevalence according to specific bodily fluid site from all studies except one (Table 1; No. 9). The highest site‐specific shedding prevalence was reported by *Faix et al.* (Table 1; No. 2) in cervical secretions, and the lowest by *Gehrz et al.* (Table 1; No.1) in urine and cervical secretions.

Only three out of nine included studies (Table 1; No. 2, 5, 9) have data on the prevalence of cCMV infection in HCMV seropositive pregnant women meeting the inclusion criteria that were extractable. *Faix et al.* (Table 1; No. 2) reported 6.3% (1/16) of cCMV infection in infants born to women with HCMV shedding detection, compared to 0% (0/29) in infants born to women with no HCMV shedding detection. *Kyriazopoulou et al.* (Table 1; No. 5), which found no HCMV shedding detection in the pregnant women tested, reported 12.5% (4/32) of cCMV infection in the foetuses (tested via amniotic fluid). *Zelini et al.* (Table 1; No. 9) reported 0% of cCMV infection in infants born to women with and without HCMV shedding detection.

### 3.5 Pooled prevalence and meta‐analyses of data in selected studies

**FIGURE 2:**
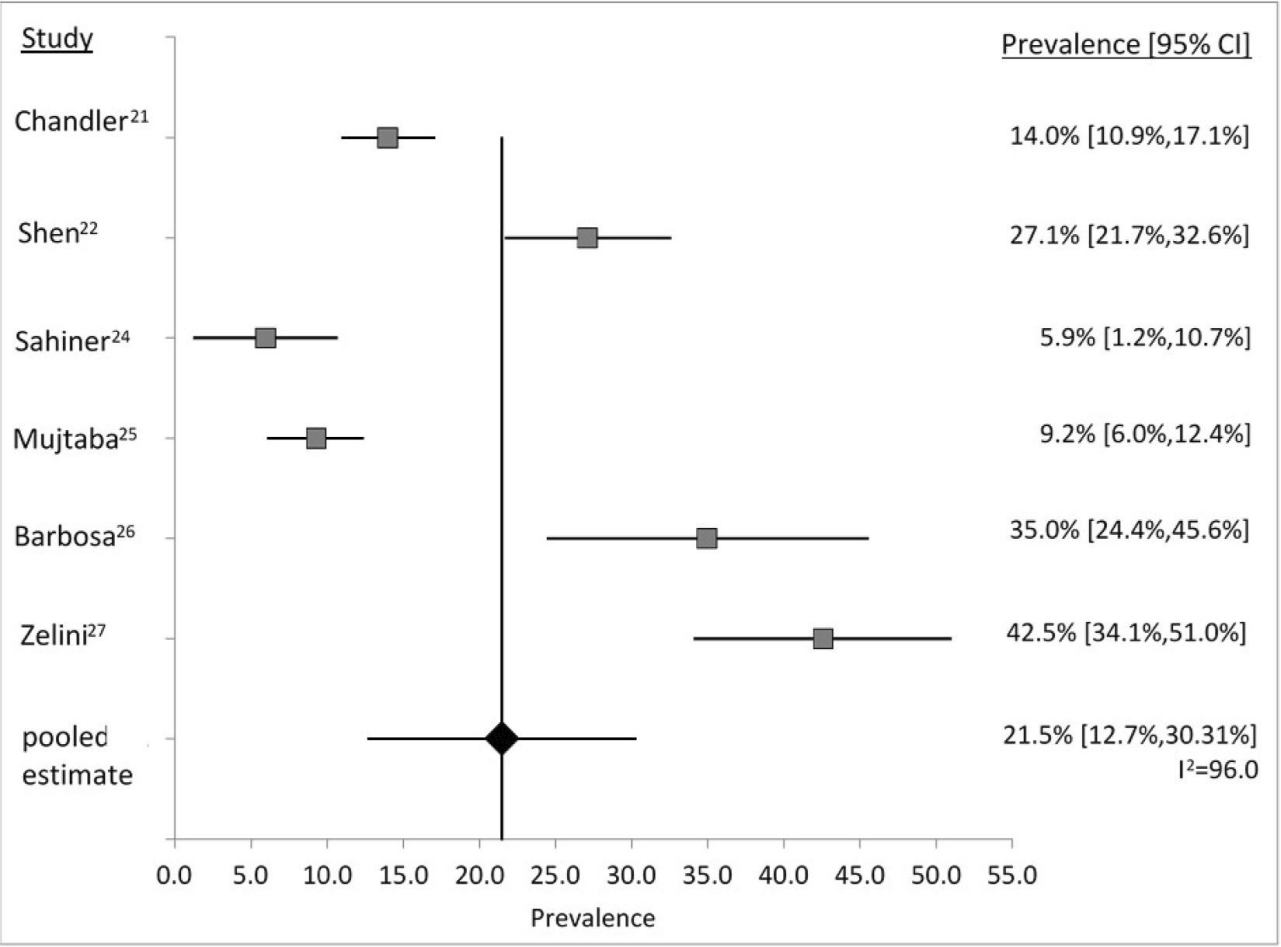
Forest plot displaying a random effects meta‐analysis of the prevalence of human cytomegalovirus (HCMV) shedding in any bodily fluid of HCMV seropositive pregnant women on selected studies. Forest plot displaying a random effects meta‐analysis of the prevalence of CMV shedding in any bodily fluid of CMV seropositive pregnant women on selected studies with suitable sample size. Each square represents the prevalence for each study included in the meta‐analysis, placed in position to the corresponding study on the y‐axis and the corresponding prevalence estimate on the y‐axis. The pooled estimate prevalence is marked by a diamond. The length of the line on either side of each square and diamond represents the extent of the prevalence 95% CI. The prevalence estimate and 95% CI for each study and for the pooled estimate are also displayed on the far right, including Higgins I^2^ score for the pooled estimate displayed as I^2^ to analyse heterogeneity across the studies.

The pooled prevalence estimate of HCMV shedding in any bodily fluids in HCMV seropositive pregnant women from the meta‐analysis performed was 21.5% [95% CI 12.7%,30.3%], as shown in Figure 2. Six studies met the sample size criteria (Table 1; No. 3, 4, 6–9). The pooled prevalence had a high level of heterogeneity among the study results (*I* ^2^ = 96.0%). The prevalence from individual studies included in the meta‐analysis ranged from 5.9% to 42.5%, such that the highest prevalence was seven times greater than the lowest prevalence. The average quality assessment score of the critical appraisal performed on the studies included in the systematic review are described in Table 1; from the six studies included in the meta‐analyses, three (Table 1; No. 3, 4, 9) had a high score, two (Table 1; No. 7, 8) a medium score and one (Table 1; No. 6) a low score.

Subgroup meta‐analyses of site‐specific prevalence of HCMV shedding in pregnant women were possible for cervicovaginal secretions (cervical and vaginal secretion estimates were grouped together), urine, and blood, with an order of highest to lowest pooled prevalence estimated in cervicovaginal secretions (18.5% [9.3%,27.7%]), urine (9.7% [4.8%,14.3]), and blood (3.9% [0%,8.5%]). All subgroup meta‐analyses had high levels of heterogeneity across the studies as measured by Higgins I^2^ (Table 2).

**TABLE 2:** Subgroup meta‐analyses of the prevalence of human cytomegalovirus (HCMV) shedding in HCMV seropositive pregnant women according to site of bodily fluid

Site of HCMV shedding	Studies included	Studies	Prevalence range	Pooled prevalence estimate (% [95% CI])	Heterogeneity (Higgins I^2^%)
Blood	3	Sahiner 2015,^24^ Mujtaba, Barbosa 2018^26^	0.8%–9.2%	3.9% [0%*,8.5%]	90.5
Cervicovaginal secretions	3	Chandler 1985,^21^ Shen 1993,^22^ Barbosa 2018^26^	12.5%–30.0%	18.5% [9.3%,27.7%]	88.3
Urine	3	Shen 1993,^22^ Sahiner 2015,^24^ Barbosa 2018^26^	5.0%–13.3%	9.7% [4.8%,14.3]	68.7

*Note*: Table detailing the subgroup meta‐analyses of the prevalence of HCMV shedding in HCMV seropositive pregnant women according to site of detection, performed on selected studies with suitable sample size, ordered alphabetically by the bodily fluid site. Prevalence of HCMV shedding in cervical and vaginal secretions found in the studies were grouped together as HCMV shedding in cervicovaginal secretions to perform the meta‐analyses. Pooled prevalence estimates are displayed with 95% CI. Higgins I^2^ score for each pooled prevalence estimate was calculated Io analyse heterogeneity across the studies. *Substituted from a negative lower CI of −0.8%.

Abbrevitaion: CI, confidence interval.

## 4 DISCUSSION

To our knowledge, our review is the first to systematically summarise the prevalence of HCMV shedding in HCMV seropositive pregnant women, in different populations around the world.

We have assembled evidence that HCMV shedding is indeed detected in HCMV seropositive women during pregnancy, up to delivery. Furthermore, we found a pooled prevalence estimate that 21.5% of HCMVseropositive pregnant women will shed HCMV on at least one time‐point during pregnancy. As HCMV shedding may suggest that an individual is acutely infected with HCMV,^1^, ^2^ HCMV shedding in a HCMV seropositive pregnant woman may suggest that she has developed a non‐primary maternal HCMV infection. It is known that there is an association between cCMV infection and non‐primary maternal HCMV infection.^28^, ^29^, ^30^, ^31^ Thus, HCMV shedding may aid in identifying HCMV seropositive pregnant women at higher risk of having an infant with cCMV infection.

According to bodily fluid site, the pooled prevalence estimate in our analysis was highest for cervicovaginal secretions, which may suggest that cervicovaginal secretions are the most suitable samples for assessing HCMV shedding in HCMV seropositive pregnant women. As the vertical transmission of HCMV from a pregnant mother to her foetus occurs either transplacentally or during vaginal delivery,^2^, ^6^ contact with HCMV‐infected cervicovaginal secretions may provide a mechanism for this.^1^, ^2^ Assessment of cervicovaginal secretions may be a practical way of identifying HCMV seropositive pregnant women with non‐primary HCMV infection.

The association between HCMV shedding in pregnant women and the vertical transmission of cCMV has been poorly investigated. We have gathered evidence here to suggest that this important gap in our knowledge needs to be addressed, and it may contribute towards the development of disease burden models and therapeutic or preventative strategies against cCMV infection.

The potential risk factors for HCMV shedding in HCMV seropositive pregnant women also needs to be investigated further. Exposure to young children was identified as a risk factor for HCMV shedding in one of the three included studies in the systematic review, that reported varying data on this. Young children commonly shed HCMV for prolonged periods of time and have been shown to be a common source of HCMV transmission to pregnant women.^1^, ^3^ Exposure to young children is likely to be a risk factor for HCMV shedding in HCMV seropositive pregnant women too, however this was not shown conclusively in all studies.

While this review aims to understand the prevalence of HCMV shedding in HCMV seropositive pregnant women in order to facilitate the estimation of disease burden, an understanding of the distribution of HCMV shedding during pregnancy up to delivery would also contribute to this. Five of the nine studies included in the systematic review adopted a longitudinal study design, which could add important information on the disease burden to contribute towards the development of preventative and therapeutic strategies for cCMV infection.

There is a paucity of published literature on the prevalence of HCMV shedding in HCMV seropositive pregnant women and this systematic review identified only nine relevant studies. As addressed, this may be due to the value of this epidemiological information being underestimated. The appraised quality of the studies included in this systematic review and the meta‐analyses also varied, few with low scores where the prevalence and pooled prevalence estimates should be interpreted with caution.

Although the studies represented a considerable range of countries and continents, only studies published in English were included, which may limit the applicability of these results internationally. Indeed, a third of the studies were from the USA, and no studies represented Africa or Australia continents. Therefore, it may be difficult to generalise our findings for populations that were not studied.

The high level of statistical heterogeneity^32^ identified across the studies included in the meta‐analyses means that the pooled prevalence estimates from the meta‐analyses should be interpreted with caution. The population demographics such as ethnicity, socio‐economic status, age and status of exposure to children also varied amongst all studies included in this systematic review. Due to this variability, it was not possible to determine the association of these factors with HCMV shedding, but it may have had an impact on the prevalence of HCMV shedding found.

## 5 CONCLUSION

To our knowledge, our review is the first to systematically search the literature to summarise the available evidence on the prevalence of HCMV shedding in HCMV seropositive pregnant women. Human cytomegalovirus shedding could aid in identifying HCMV seropositive pregnant women at an increased risk of having an infant with cCMV, and our findings may contribute towards the development of disease burden models and therapeutic or preventative strategies against cCMV infection. There is insufficient evidence at present about the global prevalence of HCMV shedding in HCMV seropositive pregnant women; more research is needed to assess this important epidemiological question in different populations.

## AUTHOR CONTRIBUTIONS

**Shari Sapuan**: Conceptualisation, Investigation, Methodology, Formal Analysis, Data Curation Resources, Writing ‐ Original Draft Preparation, Writing ‐ Review and Editing. **Anastasia A. Theodosiou**: Investigation, Methodology, Formal Analysis, Data Curation Resources, Writing ‐ Original Draft Preparation, Writing ‐ Review and Editing. **Blair L. Strang**: Supervision, Writing ‐ Original Draft Preparation, Writing ‐ Review and Editing, Project Administration, Funding. **Paul T. Heath**: Conceptualisation, Supervision, Writing ‐ Original Draft Preparation, Writing ‐ Review and Editing, Project Administration, Funding. **Christine E. Jones**: Conceptualisation, Investigation, Methodology, Formal Analysis, Data Curation Resources, Supervision, Writing ‐ Original Draft Preparation, Writing ‐ Review and Editing, Project Administration.

## CONFLICT OF INTEREST

The authors have no competing interest.

## Supporting information

Supplementary Material

## Data Availability

The data that supports the findings of this study are available within this manuscript, its supplementary materials, and from the corresponding author upon reasonable request.
