# Supplementary material for: A systematic review and meta‐analysis of the prevalence of human cytomegalovirus shedding in seropositive pregnant women
Source: Rev Med Virol. 2022 Oct 5;32(6):e2399. doi: 10.1002/rmv.2399 (PMC9786761; doi:10.1002/rmv.2399)
Supplement: Supplementary file 1 — Supplementary Material [file RMV-32-e2399-s001.docx]

**A systematic review and meta-analysis of the prevalence of human cytomegalovirus shedding in seropositive pregnant women**

**SUPPLEMENTARY MATERIAL**

**Search strategies used in the systematic review for different databases**

Database: Ovid MEDLINE® and In-Process, In-Data-Review & Other Non-Indexed Citations, 1946 to 06/12/21 (Accessed on 07/12/21)

Search Strategy:

1 cmv.ti. (3338)

2 cytomegalovirus.ti. (23897)

3 shed*.tw. (100233)

4 excret*.tw. (177052)

5 replicat*.tw. (307142)

6 dna.tw. (1058718)

7 pcr.tw. (538521)

8 culture.tw. (607491)

9 seropositiv*.tw. (40287)

10 igg.tw. (142743)

11 pregnan*.tw. (525404)

12 matern*.tw. (290291)

13 exp Cytomegalovirus/ (21607)

14 exp virus shedding/ (3869)

15 exp pregnant woman/ (10143)

16 1 or 2 or 13 (32682)

17 3 or 4 or 5 or 6 or 7 or 8 or 14 (2447886)

18 9 or 10 (177111)

19 11 or 12 or 15 (692267)

20 16 and 17 and 18 and 19 (185)

Database: Embase 1974 to 2021 Week 33 (accessed on 07/12/21)

Search Strategy:

1 cmv.ti. (6044)

2 cytomegalovirus.ti. (28079)

3 shed*.tw. (119671)

4 excret*.tw. (199564)

5 replicat*.tw. (367776)

6 dna.tw. (1260290)

7 pcr.tw. (778640)

8 culture.tw. (760810)

9 seropositiv*.tw. (50883)

10 igg.tw. (194568)

11 pregnan*.tw. (675147)

12 matern*.tw. (375166)

13 exp Cytomegalovirus/ (42232)

14 exp virus shedding/ (8021)

15 exp pregnant woman/ (90110)

16 1 or 2 or 13 (55181)

17 3 or 4 or 5 or 6 or 7 or 8 or 14 (3040571)

18 9 or 10 (237359)

19 11 or 12 or 15 (878644)

20 16 and 17 and 18 and 19 (337)

Database: Web of Science Core Collection (accessed on 07/12/21)

Search Strategy:

(TI=(cmv) OR TI=(cytomegalovirus)) AND (TS=(shed*) OR TS=(excret*) OR TS=(DNA) OR TS=(PCR) OR TS=(culture)) AND (TS=(seropositive) OR TS=(IgG)) AND (TS=(pregnan*) OR TS=(maternal)) (187)
